# Addition of Prebiotics to the Ketogenic Diet Improves Metabolic Profile but Does Not Affect Seizures in a Rodent Model of Infantile Spasms Syndrome

**DOI:** 10.3390/nu14112210

**Published:** 2022-05-26

**Authors:** Chunlong Mu, Angela Pochakom, Raylene A. Reimer, Anamika Choudhary, Melinda Wang, Jong M. Rho, Morris H. Scantlebury, Jane Shearer

**Affiliations:** 1 Department of Biochemistry and Molecular Biology, Cumming School of Medicine, University of Calgary, 3330 Hospital Drive NW, Calgary, AB T2N 4N1, Canada; angela.pochakom@ucalgary.ca (A.P.); reimer@ucalgary.ca (R.A.R.); jshearer@ucalgary.ca (J.S.); 2 Faculty of Kinesiology, University of Calgary, 2500 University Drive NW, Calgary, AB T2N 1N4, Canada; 3 Alberta Children’s Hospital Research Institute, Cumming School of Medicine, University of Calgary, 3330 Hospital Drive NW, Calgary, AB T2N 4N1, Canada; anamika.choudhary@ucalgary.ca (A.C.); melinda.wang@ucalgary.ca (M.W.); morris.scantlebury@albertahealthservices.ca (M.H.S.); 4 Department of Pediatrics, Cumming School of Medicine, University of Calgary, 3330 Hospital Drive NW, Calgary, AB T2N 4N1, Canada; 5 Departments of Neurosciences, Pediatrics and Pharmacology, University of California San Diego, 9500 Gilman Drive, La Jolla, CA 92093, USA; jrho@health.ucsd.edu; 6 Department of Clinical Neurosciences, Hotchkiss Brain Institute, Cumming School of Medicine, University of Calgary, 3330 Hospital Drive NW, Calgary, AB T2N 4N1, Canada

**Keywords:** ketogenic diet, nutrition, epilepsy, metabolism, prebiotic fiber

## Abstract

The ketogenic diet (KD) is an effective treatment for infantile spasms syndrome (IS). However, the KD has implications for somatic growth, development, and the gut microbiota. The impact of incorporating a prebiotic fiber (PRE, oligofructose-enriched inulin, 0.8 g/dL) into a KD diet on spasms, developmental milestones, fecal gut microbiota, metabolites, and hippocampal mitochondrial metabolism were examined. Following IS induction, animals were randomized to KD or KD + PRE diets. A third group without IS and suckled by dams was included as a normally developing reference group (R). PRE inclusion decreased ketones and increased circulating glucose levels but had no impact on spasms. In the liver, PRE increased triglyceride concentrations, decreased carnitine levels, and downregulated genes encoding enzymes responsible for ketogenesis. In the hippocampus, PRE increased glutathione levels but did not affect the maximal respiratory capacity of mitochondria. Analysis of the gut microbiota showed that KD + PRE increased microbial richness and the relative abundance of *Bifidobacterium pseudolongum* and *Lactobacillus johnsonii*. No differences in developmental milestones (i.e., surface righting, negative geotaxis, and open field behavior) were observed between KD and KD + PRE, except for ultrasonic vocalizations that were more frequent in KD + PRE. In summary, PRE did not impact spasms or developmental outcomes, but was effective in improving both metabolic parameters and gut microbiota diversity.

## 1. Introduction

The ketogenic diet (KD) is a high-fat, low-carbohydrate, normal protein diet that is often used to treat drug-resistant epilepsy. The efficacy of the diet is well-established, with multicenter and randomized control trials showing that ~50% of patients respond to the KD (>50% reduction in seizures), with 25–30% experiencing a >90% reduction in seizures and half of the latter being seizure-free [1]. Furthermore, there is an established protocol for the implementation and management of pediatric and adult patients on the diet that is used in many clinics worldwide [2]. At present, the mechanisms underlying the beneficial impacts of the diet are unclear. However, recent work implicates the gut microbiota, the trillions of microorganisms that inhabit our intestines as playing a fundamental role [3,4].

When administered, the KD has profound impacts on the gut microbiota, as it generally lacks microbiota-accessible carbohydrates that support the growth of microbes [5,6]. Employing antibiotics and fecal microbiota transfer into germ-free mice, Olson and colleagues [7] demonstrated that the seizure-reducing effects of the diet are dependent on the gut microbiota in two rodent models of epilepsy. Likewise, gut-microbiome-based manipulations, including the KD, antibiotics, and probiotics, have been shown to suppress seizures (spasms) in a neonatal brain injury rat model of infantile spasms [3,8,9]. Similar to the abovementioned study, the protective impacts of the diet could also be transferred to another animal by fecal transplant alone, showing the gut-based dependency of the treatment [3].

In addition to the gut microbiota, the KD is known to have numerous side effects [10,11,12]. Common complaints often include gastrointestinal upset (nausea, diarrhea, vomiting, discomfort, and constipation), as well as longer-term metabolic impacts, including dyslipidemia [13]. Dyslipidemia is reported in 16–30% of patients following KD initiation [11,14,15], with cholelithiasis and hepatosteatosis also reported in older children [12]. A potential approach to mitigate these side effects may be the co-administration of prebiotic fiber. Prebiotic fibers, such as oligofructose and inulin, are nondigestible carbohydrates that are preferentially fermented by specific gut bacteria. They are generally considered beneficial for the host with positive effects on numerous aspects of metabolic health, including lipid homeostasis, reduced inflammation, and increased bifidobacteria abundance in the gut [16].

The purpose of the present study was to examine the impact of adding a prebiotic (oligofructose-enriched inulin) to a KD formula in a validated rodent model of infantile spasms [17]. Infantile spasms syndrome (IS), or West syndrome, is a type of childhood epilepsy that typically occurs in the first year of life, characterized by interictal hypsarrhythmia (EEG), flexion/extension spasms (seizures), and poor developmental outcomes [18,19,20]. First-line intervention for IS is pharmacological treatment (adrenocorticotropic hormone (ACTH), prednisolone, and vigabatrin); however, a significant number of IS cases (30–40%) are refractory to pharmacological treatment [21,22]. In these cases, the KD is administered where it is efficacious in approximately two thirds of patients [11]. Measures of interest included spasm frequency, circulating metabolites, metabolism, developmental milestones, and the gut microbiota relative to the KD alone. As seizure severity is a key predictor of neurodevelopmental impairment in IS [23], we assessed behavioral readouts associated with developmental milestones in our animal model. Metabolic and metabolomics analyses were performed in both blood and brain to assess the potential effects of the KD on dyslipidemia and mitochondrial function. Lastly, we evaluated the impacts of the prebiotic on gut microbial composition and the gut–brain axis. We hypothesized that the inclusion of prebiotic fiber would not impact KD-induced seizure protection but would improve metabolic parameters and diversify the developing gut microbiota.

## 2. Materials and Methods

### 2.1. Animals

**Figure 1:**
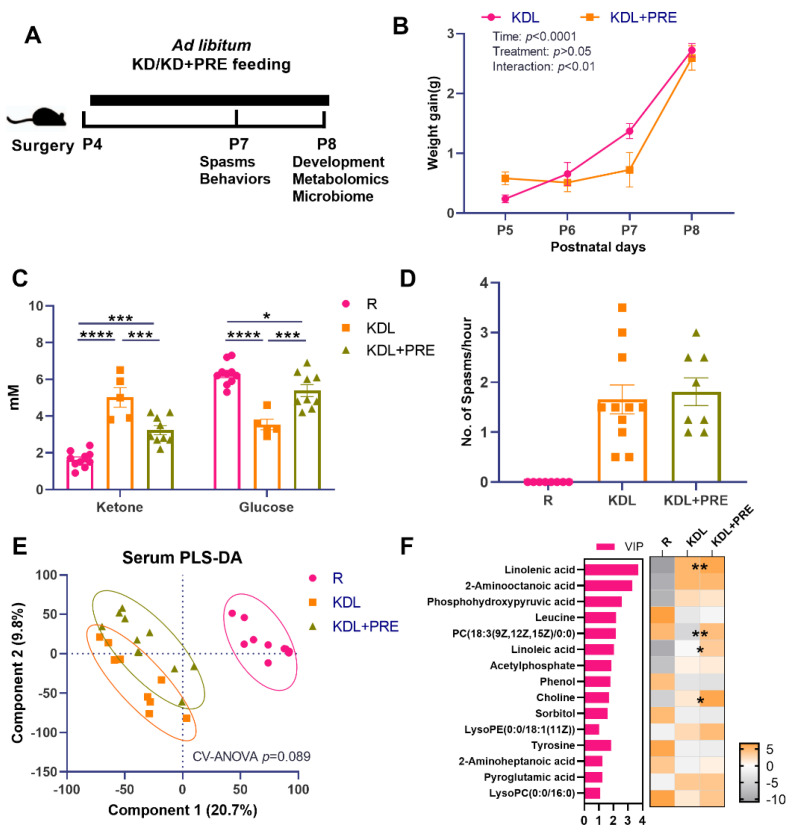
Effects of prebiotic inclusion on body mass, seizures, and systemic metabolism. (**A**) The experimental design. (**B**) Body weight gain. (**C**) Blood ketone and glucose concentrations. (**D**) Spasms frequency. (**E**) PLS-DA analysis of serum metabolites. (**F**) Discriminant metabolites with *p* < 0.05 and VIP > 1 in the serum. In the heatmap, * *p* < 0.05, ** *p* < 0.01 comparing KDL vs. KDL+PRE. R-normally developing reference group; KDL-ketogenic diet formula; KDL+PRE-ketogenic diet formula plus prebiotic fiber. Data are presented as mean ± SEM with individual values (**B**–**D**). *n* = 6–13 rats/group. * *p* < 0.05, *** *p* < 0.001, **** *p* < 0.0001.

All experimental procedures were approved by the Health Sciences Animal Care Committee at the University of Calgary in accordance with the guidelines set forth by the Canadian Council on Animal Care (protocol #AC20-0013). All animal experiments were conducted on offspring of timed pregnant Sprague-Dawley rats (Charles River Laboratories, St. Constant, QC, Canada). Sprague-Dawley females were bred in-house, in full compliance with the ethical protocol. Animals were housed at a constant temperature (20–22 °C) on a 12-h light/dark cycle at the specific pathogen-free facility of the Health Sciences Animal Resource Centre, University of Calgary, Canada. Animals were randomized into three experimental settings (Figure 1A): (1) a normally developing reference group (R; *n* = 13) wherein animals were kept with their mother and fed breast milk. Surgery was not performed in this reference control group; (2) ketogenic diet with lesion-induced epilepsy (KDL; *n* = 9); and (3) ketogenic diet with lesion-induced epilepsy plus prebiotic fiber (oligofructose-enriched inulin, PRE; *n* = 11) at 0.8 g/dL. As the objective of this study was to examine the impact of PRE in the context of the KD, additional groups including PRE alone, normal carbohydrate, and normal carbohydrate+PRE were not included. The addition of PRE to infant diets has been widely studied and has been shown to cause increases in *Bifidobacterium* in both animal models and humans [24,25].

Data regarding the impact of normal carbohydrate and the KD on spasms, metabolites, and the gut microbiota have been previously published [3,8,26]. Both male and female animals were used for the study. The induction of IS and animal rearing were performed as previously described [17,26]. Briefly, at postnatal day (P) 4, male and female neonatal rats were positioned in a stereotaxic apparatus for neonatal rat surgery (Benchmark Angle One, MyNeurolab.com, St. Louis, MO, USA). After hypothermic anesthesia, doxorubicin was injected into the right lateral ventricle at 5 µg/2.5 µL, and lipopolysaccharide into the right parietal cortex at 3 µg/1.5 µL. The co-ordinates used were as follows: doxorubicin, 2.68 mm anterior to lambda, 1.1 mm lateral to sagittal suture, 3.3 mm deep; lipopolysaccharide, 2.55 mm anterior to lambda, 1 mm lateral to sagittal suture, 1.7 mm deep. In the morning of P5, animals were given an intraperitoneal injection with 200 mg/kg p-chlorophenylalanine. This ‘triple-hit model’ is a validated model of symptomatic infantile spasms that mimics many human characteristics of IS [17]. The two IS groups were artificially reared once the animals fully recovered from the anesthesia. Animals were individually placed in Styrofoam cups warmed in a water bath (45 °C) and filled with bedding material (31–33 °C). Animals were continuously fed either a KD (4:1 ratio by weight of fats to carbohydrate plus protein, Table S1) with or without PRE from P5 to P8 through an implanted cheek cannula, as previously described [26]. The time-frame was chosen as this is a period when animals experience a high rate of seizures and when the antiseizure effects of the KD mimics what is observed in infants with seizure reductions in the range of 50% [26]. All experiments were performed with the aim of keeping animal pain, stress, and numbers to a minimum, as stated in our ethics certification. Colonic contents, liver, serum, and hippocampus were collected, snap frozen, and stored at −80 °C for microbiota and metabolism analysis.

### 2.2. Spasms/Seizure Quantification

Spasms characterized by sudden extensor and flexor movements were recorded using a video system with Sirenia software (Pinnacle Technology, Lawrence, KS, USA) following previously described methods [17,26]. A total of 4 h of recordings on P7 were quantified per animal. Spasms were quantified by date and by a single reviewer who was blinded to the treatments.

### 2.3. Developmental Milestones

As developmental milestones are impaired in animals with neurodevelopmental deficits [27] and have been previously shown to improve with the KD [26], we assessed vocalization, surface righting, negative geotaxis, and open field testing on P7 and P8, as previously described [26].

### 2.4. Blood and Serum Measurements

Hepatic triglycerides were measured according to the manufacturer’s instructions (Cayman Chemical Company, Ann Arbor, MI). Blood glucose and ketone concentrations were measured at euthanasia at P8 with a FreeStyle Precision Neo meter (Abbott Laboratories, Abbott Park, IL, USA).

### 2.5. Total RNA Extraction and Real-Time q-PCR

Total RNA was extracted using AllPrep DNA/RNA Kits (Qiagen) RT-qPCR, as previously described [28]. Tyrosine 3-monooxygenase/tryptophan, 5-monooxygenase activation protein, zeta polypeptide (*Ywhaz*), and Cyclophilin A (*CycA*) were used as housekeeping genes. The relative gene expression of AMP-activated protein kinase (*Ampk*), acetyl-CoA acetyltransferase 1 (*Acat1*), fatty acid synthase (*Fas*), 3-hydroxy-3-methylglutaryl-CoA synthase 2 (*Hmgcs2*), and carnitine palmitoyltransferase 1A (*Cpt1α*) were measured using the primers listed in Table S2.

### 2.6. DNA Extraction and 16S rRNA Gene Sequencing

Total genomic DNA was extracted from colonic contents using QIAamp DNA kit (Qiagen Ltd. GmbH, Germany) per the manufacturer’s instructions. DNA purity was checked with a Nanodrop 2000 Spectrophotometer (Thermo Fisher Scientific, Waltham, MA, USA). The concentration was quantified using a high-sensitivity dsDNA Qubit Kit (Invitrogen, Carlsbad, CA, USA). The extracted DNA was stored at −80 °C for further analysis. Bacterial 16S rRNA genes were polymerase chain reaction (PCR)-amplified using dual-barcoded primers targeting the V4 region (515F 5′-GTGCCAGCMGCCGCGGTAA-3′, and 806R 5′-GGACTACHVGGGTWTCTAAT-3′) according to the MiSeq protocol [29]. The 300-bp paired-end sequencing reaction was performed on an Illumina MiSeq platform in the Microbiome Insights laboratory in Vancouver (Richmond, BC, Canada). Sequences were denoised and classified using Greengenes version 13-8 as the reference database and assigned into operational taxonomic units (OTUs) at 97% similarity using the Mothur software (v1.45.3) [29] following the protocol (https://mothur.org/wiki/miseq_sop/, accessed on 12 July 2021).

### 2.7. Mitochondrial Respirometry

High-resolution respirometry was performed on hippocampal samples from the side contralateral to the lesion using an OROBOROS Oxygraph-2k (Oroboros Instruments, Innsbruck, Austria), as previously described [30,31]. Following brain removal, hippocampus tissue was quickly isolated and weighed at 2 mg and transferred into calibrated Oxygraph-2k 2 mL chambers containing MiR05 respiration medium (37 °C). Oxygen concentration (μM), as well as oxygen flux per tissue mass (pmol O_2_·s^−1^·mg^−1^), were recorded in real time using DatLab software (Oroboros Instruments, Innsbruck, Austria). Samples were permeabilized with 50 µg mL^−1^ saponin, and the substrate-inhibitor-uncoupled titration protocols were as previously described [32]. Pyruvate (5 mM), malate (0.5 mM), and glutamate (10 mM) were injected to evaluate the non-phosphorylating LEAK respiration (CI*_L_*). OXPHOS capacity of CI-linked activity (CI*_P_*) was measured after addition of ADP (2.5 mM). Cytochrome C of 0.01 mM was added for quality control analysis of outer mitochondrial membrane integrity. Then, OXPHOS capacity with combined CI and II-linked substrates (CI+CII*_P_*) was assessed by addition of succinate (10 mM). Uncoupled respiration was determined by stepwise titration of 0.5mM carbonylcyanide p-trifluoromethoxyphenylhydrazone (FCCP) until maximum respiration was achieved, which was used as a measurement of the maximum capacity of the electron transfer system (ETS). Subsequent inhibition of CI and CIII by rotenone (0.05 µM) and antimycin A (2.5 µM), respectively, was used to measure nonmitochondrial respiration, which was subtracted from every mitochondrial respiratory state.

### 2.8. Metabolomics Profiling

A total of 50 μL of serum, ~20 mg of liver, and ~10 mg of hippocampus tissues were used for sample preparation following previously reported methods [33,34]. A total of 2 μL of extracts was injected to an Agilent 6550 iFunnel Q-TOF LC/MS instrument (Agilent, Santa Clara, CA, USA) with an Acquity UPLC HSS T3 column [35]. Ions were analyzed in positive ion mode. Raw Agilent data were transformed to mzXML file by ProteoWizard 3.0 and uploaded to XCMS online for analysis [36]. Compounds were identified with METLIN [37] and Human Metabolome Database [38]. Data normalization and statistical analysis, including partial least-squares discriminant analysis (PLS-DA) and variable importance in projection (VIP) analysis, were performed with Metaboanalyst 4.0 [39]. Discriminant metabolites were defined with FDR-adjusted *p*-value < 0.05.

### 2.9. Statistical Analysis

Data are presented as mean ± SEM. Comparisons between two groups were conducted using a nonparametric Mann-Whitney U test or Student’s *t* test, depending on the normality of data distribution by Bartlett’s test. One-way ANOVA with Tukey’s post hoc was used to determine differences between groups. Comparisons of bacteria taxa were analyzed by Kruskal-Wallis ANOVA with Dunn’s post hoc test (GraphPad Software, San Diego, CA, USA). A false discovery rate correction was applied for multiple tests. Significant differences are indicated by * *p* < 0.05, ** *p* < 0.01, *** *p* < 0.001, and **** *p* < 0.0001.

## 3. Results

### 3.1. Animal Characteristics and Seizure Frequency

A schematic of the key outcome measures is shown in Figure 1A. There was no difference between KDL and KDL+PRE groups in terms of weight gain (*p* = 0.443, Figure 1B). In the blood, the KD significantly increased the concentration of ketones and decreased that of glucose compared to the R group (*p* < 0.0001, Figure 1C). These changes were reversed in the KDL+PRE rats. Compared to the KDL group, the KDL+PRE group had significantly decreased concentrations of ketone and increased glucose (*p* < 0.001, Figure 1C).

As ketones are thought to mediate the antiseizure impacts of the KD [40], we assessed spasms in the animals. In our model, the number of spasms is a basic readout of IS severity. Previous studies by Choudhary et al. [26] have shown that, when IS animals are placed on a KD, spasms are reduced by ~50% compared to those on a carbohydrate-containing diet. The KDL+PRE did not change the spasms frequency relative to KD (*p* = 0.665, Figure 1D), despite lowering ketone and elevating glucose levels (Figure 1C). Serum metabolomics profiling was then conducted to investigate whether KDL+PRE affected systemic metabolism. We observed a distinct sample distribution of KD compared to R treatment (Figure 1E), showing the KD changed overall metabolite composition. The samples from KDL and KDL+PRE also clustered separately (Figure 1E). Among the discriminant metabolites (*p* < 0.05, VIP > 1), KDL+PRE significantly affected metabolites, including linolenic acid (VIP = 3.72) and choline (VIP = 1.68, Figure 1F), both of which can be generated via the gut microbiota [41,42].

### 3.2. Developmental Milestones and Communication

**Figure 2:**
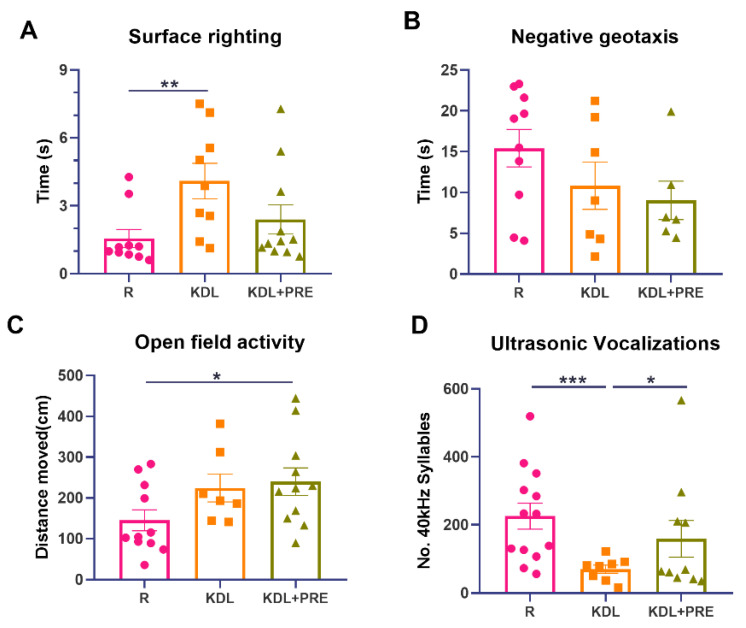
Developmental milestones and communication measurements. (**A**) Surface righting times. (**B**) Negative geotaxis activity. (**C**) Open field activity. (**D**) Ultrasonic vocalizations. R-normally developing reference group; KDL-ketogenic diet formula; KDL+PRE-ketogenic diet formula plus prebiotic fiber. Data are presented as mean ± SEM with individual values. *n* = 6–13 rats/group. * *p* < 0.05, ** *p* < 0.01, *** *p* < 0.001.

To uncover any impact of PRE on developmental milestones, four tests were performed. The KD, but not KDL+PRE, resulted in impaired surface righting (*p* = 0.009) (Figure 2A). No statistical difference was observed for negative geotaxis (one-way ANOVA *p* = 0.193, Figure 2B), although both surface righting (*p* = 0.065, KDL vs. KDL+PRE) and negative geotaxis (*p* = 0.653, KDL vs. KDL+PRE) were improved with PRE. For open field, both KDL and KDL+PRE led to greater locomotion compared with the R group (*p* = 0.105 for R vs. KDL, *p* = 0.031 for R vs. KDL+PRE, Figure 2C). Communication was assessed by ultrasonic vocalization. The KD decreased the number of 40 kHz syllables (*p* < 0.001) compared to the R group. When compared to the KDL group, the inclusion of PRE increased the number of 40 kHz syllables (*p* = 0.040) to a level similar to the R animals (Figure 2D). In summary, the KDL+PRE partially improved developmental milestones compared to KDL, by increasing the open field locomotion and enhancing the number of ultrasonic vocalizations.

### 3.3. Metabolic Alterations by Prebiotic Fiber Inclusion

**Figure 3:**
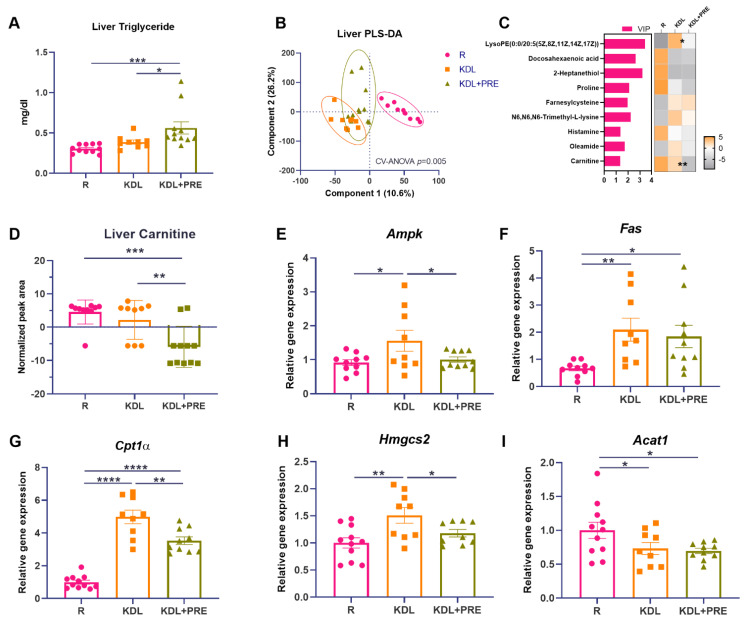
Effects of the prebiotic inclusion on liver metabolism. (**A**) Liver triglyceride. (**B**) PLS-DA analysis of liver metabolites. (**C**) Discriminant metabolites with *p* < 0.05 and VIP > 1 in the liver. In the heatmap, * *p* < 0.05, ** *p* < 0.01 comparing KDL vs. KDL+PRE. (**D**) Liver carnitine. (**E**–**I**) Gene expression of enzymes related to lipid metabolism in the liver. Data are presented as mean ± SEM with individual values. *Acat1*-acetyl-CoA acetyltransferase 1; *Ampk*-AMP-activated protein kinase; *Cpt1α*-carnitine palmitoyltransferase 1A; *Fas*-fatty acid synthase; *Hmgcs2*-3-Hydroxy-3-Methylglutaryl-CoA synthase 2; R-normally developing reference group; KDL-ketogenic diet formula; KDL+PRE-ketogenic diet formula plus prebiotic fiber. *n* = 9–11 rats/group. * *p* < 0.05, ** *p* < 0.01, *** *p* < 0.001, **** *p* < 0.0001.

The liver is the major site responsible for ketone production, and ketones are thought to mediate, in part, the antiseizure efficacy of the KD [43]. In the liver, the triglyceride levels were increased by PRE inclusion, compared to both R (*p* < 0.001) and KDL groups (*p* = 0.021, Figure 3A). The metabolomics profiling of liver homogenates was then performed to evaluate metabolic alterations. Samples from different treatment groups showed distinct metabolite clusters (CV-ANOVA, *p* = 0.005, Figure 3B). Comparing the discriminant metabolites (*p* < 0.05, VIP > 1, Figure 3C), the relative concentrations of lysophosphatidylethanolamine (lysoPE), docosahexaenoic acid, and carnitine were decreased in KDL+PRE relative to KDL. Of interest, carnitine, a major metabolite that contributes to lipid oxidation, was decreased (*p* < 0.001 for R vs. KDL+PRE, *p* = 0.002 for KDL vs. KDL+PRE, Figure 3D).

To investigate the potential mechanisms accounting for the accumulation of triglycerides, we assessed the gene expression of key enzymes related to lipid metabolism. In the liver, the KDL upregulated the mRNA expression of *Ampk* compared to R animals (*p* = 0.019), which was reversed by PRE inclusion (*p* = 0.041, Figure 3E). The mRNA expression of *Fas* was also upregulated by the KDL (*p* = 0.007) compared to the R animals, but was not affected by PRE inclusion (*p* = 0.611, KDL vs. KDL+PRE, Figure 3F). *Cpt1α*, a rate-limiting enzyme of lipid oxidation, was upregulated by the KDL (*p* < 0.001) compared to R, which was reversed by PRE inclusion in the KDL (*p* = 0.002, KDL vs. KDL+PRE, Figure 3G). A similar alteration was also observed for the mRNA expression of *Hmgcs2* (Figure 3H), a rate-limiting enzyme of ketogenesis. PRE inclusion in the KDL showed the lowest expression of *Acat1* (*p* = 0.024, R vs. KDL+PRE, Figure 3I), an enzyme involved in ketogenesis. Collectively, these results indicate a downregulation of lipid oxidation and ketogenesis in the liver with KDL+PRE that may contribute to the decrease in ketones in the circulation.

### 3.4. Hippocampal Metabolites

**Figure 4:**
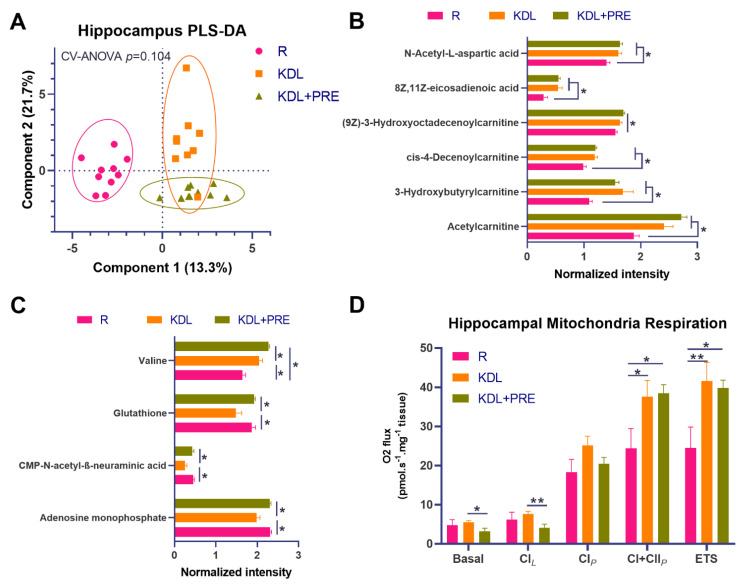
Effects of prebiotic inclusion on the metabolic phenotype and mitochondrial respiration in the hippocampus. (**A**) PLS-DA analysis of hippocampal metabolites. (**B**) Metabolites with significant changes in both KDL and KDL+PRE groups relative to R control group. (**C**) Metabolites with significant changes in KDL+PRE group against KDL group. (**D**) Mitochondrial respiratory measurements as follows; Basal, basal respiration; CI*_L_*, non-phosphorylating LEAK respiration; CI*_P_*, OXPHOS capacity of CI-linked activity; CI+CII*_P_*, OXPHOS capacity with combined CI and II-linked substrates; ETS, maximum capacity of the electron transfer system. R-normally developing reference group; KDL-ketogenic diet formula; KDL+PRE-ketogenic diet formula plus prebiotic fiber. Data are presented as mean ± SEM. *n* = 9–11 rats/group. * *p* < 0.05, ** *p* < 0.01.

To better understand mediators of the gut–microbiota–brain axis, high-resolution metabolomics profiling was conducted on hippocampal tissues. Results showed distinct metabolite composition between groups (Figure 4A). The KD alone or in combination with PRE increased the relative concentrations of carnitines, including acetylcarnitine, 3-hydroxybutyrylcarnitine, cis-4-decenoylcarnitine, and (9Z)-3-Hydroxyoctadecenoylcarnitine compared to R (*p* < 0.05), corresponding to an increase in eicosadienoic acid (*p* < 0.05, Figure 4B). Relative to KDL, the KDL+PRE increased the concentrations of glutathione (*p* = 0.002), CMP-*N*-acetyl-β-neuraminic acid (*p* = 0.004), and adenosine monophosphate (*p* < 0.001, Figure 4C), with no differences observed between R and KDL with PRE inclusion for these metabolites (*p* = 0.676, 0.550, 0.881 for glutathione, CMP-*N*-acetyl-β-neuraminic acid, and adenosine monophosphate, respectively).

As both hippocampal excitability and mitochondrial dysfunction are implicated in seizures [44], we tested mitochondrial function in the hippocampus using high-resolution respirometry. Compared to R animals, KD rats had increased phosphorylation-linked oxygen respiration in complex I and II, whether alone or in combination with PRE (*p* < 0.05, Figure 4D). When compared to KDL, the KDL+PRE decreased basic respiration (*p* = 0.034) and leak respiration in complex I (*p* = 0.009, Figure 4D).

### 3.5. Colonic Microbiota Composition

**Figure 5:**
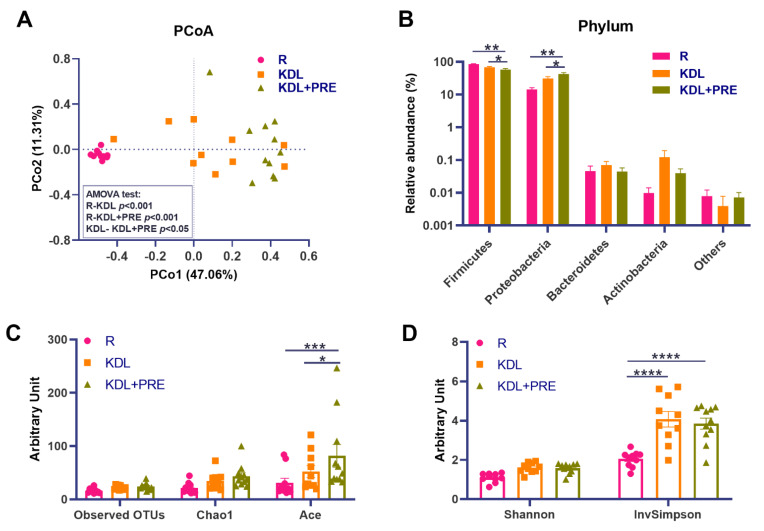
Effects of prebiotic inclusion on the microbiota community structure. (**A**) Principal co-ordinates analysis of microbial structures. (**B**) Phylum-level composition. α-diversity parameters (Shannon and InvSimpson indices). (**C**,**D**) α-diversity parameters (observed OTUs, Chao1, and Ace indices). R-normally developing reference group; KDL-ketogenic diet formula; KDL+PRE-ketogenic diet formula plus prebiotic fiber; OTU-operational taxonomic unit; PCoA-principal co-ordinates analysis. Data are presented as mean ± SEM. *n* = 10–11 rats/group. * *p* < 0.05, ** *p* < 0.01, *** *p* < 0.001, **** *p* < 0.0001.

PRE is known to benefit the gut environment by altering the gut microbiota [45]. To assess this in our model, we conducted high-throughput 16S rRNA gene sequencing on the colonic digesta of animals. Comparisons of the microbial community structure across groups showed distinct distributions as shown by a PCoA plot (Figure 5A). An AMOVA test showed the pair-wise significance (*p* < 0.05) of microbial structures between the KDL and KDL+PRE groups (Figure 5A), supporting the significant influence of PRE inclusion on the gut microbiota. At the phylum level, the KDL+PRE decreased the relative abundance of Firmicutes and increased those of Proteobacteria compared to the R and KDL groups (*p* < 0.05, Figure 5B). KDL+PRE had a higher microbial richness than R, as shown by the ACE (*p* < 0.001, Figure 5C) and InvSimpson indices (*p* < 0.001, Figure 5D).

**Figure 6:**
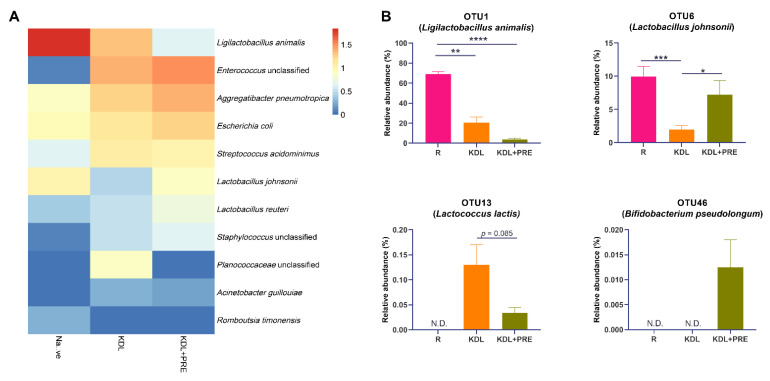
Effects of prebiotic inclusion on gut microbiota composition. (**A**) Heatmap of the predominant species. Data are presented as means. (**B**) Species with significant alterations. R-normally developing reference group; KDL-ketogenic diet formula; KDL+PRE-ketogenic diet formula plus prebiotic fiber. Data are presented as mean ± SEM. *n* = 10–11 rats/group. * *p* < 0.05, ** *p* < 0.01, *** *p* < 0.001, **** *p* < 0.0001.

At the OTU level, *Ligilactobacillus animalis*, *Enterococcus* unclassified, *Aggregatibacter pneumotropica*, *Escherichia coli*, *Streptococcus acidominimus*, *Lactobacillus johnsonii*, and *Lactobacillus reuteri* were the predominant species (Figure 6A). A detailed list of the detected OTUs can be found in Table S3. Compared to the KDL group, PRE inclusion in the KDL decreased the relative abundance of OTU1 (*Ligilactobacillus animalis*, *p* = 0.124) and OTU13 (*Lactococcus lactis*, *p* = 0.085), while increasing OTU6 (*Lactobacillus johnsonii*, *p* = 0.020) (Figure 6B). PRE inclusion enriched OTU46 (*Bifidobacterium pesudolongum*), which was not present in R and KDL animals.

## 4. Discussion

When antiseizure medications fail, clinicians often turn to the KD as a treatment option [14]. In infants, this is accomplished by the administration of a special KD formula that is often fed by an enteral route, usually in a 3:1 or 4:1 formulation [46]. Consisting of high fat, low carbohydrate, and normal protein intake, the diet has been shown to be equally effective to ACTH, a common medication in controlling seizures, and better tolerated in IS over a six-month period [14]. While effective in reducing seizures, the KD can have many side effects. For this reason, we explored the potential of PRE as an adjunct to KD therapy.

Results show that inclusion of PRE as a part of the KD did not affect spasm frequency but did reverse some KD-induced alterations, including a decrease in blood ketones and a corresponding increase in blood glucose. This is important as it suggests that the antiseizure effects of the KD are not solely dependent on circulating ketones. Indeed, inhibition of glycolysis through administration of 2-deoxyglucose or the augmentation of ketogenesis through β-hydroxybutyrate administration fail to reduce spasms in the betamethasone-*N*-methyl-d-aspartate (NMDA) model of rat IS [47], although the opposite effect has also been reported [48]. Additional KD-related mechanisms may include the alteration in gut-derived tryptophan metabolites [3] or dietary-induced cerebral acidosis [26]. Regardless of the mechanisms, our results show that, although lowered, levels of ketones with PRE were sufficient to maintain the antiseizure effects of the diet.

IS is known to be associated with poor neurodevelopmental outcomes. As PRE administration has been previously reported to impact behavior [49,50], we evaluated a battery of four behavioral tests. Results demonstrated no change between the KD and KD+PRE for surface righting, negative geotaxis, or open field activity. However, we did note alterations in ultrasonic vocalizations. Rodents have a rich repertoire of ultrasonic vocalizations reflecting the states of depression, fear, or anxiety [51]. Animals on the KD had lower levels of vocalization that were significantly increased with PRE treatment. The cause of increased vocalizations with PRE is not known, but the same phenomenon has been shown in the BTBR mouse model of autism where PRE increased total calls, call duration, and syllable repertoire with supplementation.

While there are multiple benefits reported with PRE supplementation, one effect of high relevance to the KD includes its impacts on hepatosteatosis [52,53]. PRE supplementation has been routinely found to improve nonalcoholic fatty liver disease by reducing liver enzymes and improving lipid profiles [54,55]. To this end, we assessed key markers and regulators of lipid metabolism. Compared to reference animals, the KD increased liver triglyceride content. This increase was further exaggerated with PRE supplementation. Generally, PRE supplementation is associated with either a decrease or no change in liver triglycerides in animal studies when added to chow or high-fat/high-sucrose diets [56,57], so an increase with the KD was somewhat unexpected. To better understand this finding, we examined liver carnitines as well as key genes involved in liver metabolism. Carnitine is essential for the transport of long-chain fatty acids to the mitochondrial matrix for β-oxidation. The decreased carnitine level in PRE relative to the KD suggests a reduced fatty acid metabolism. To further examine the impact of PRE on metabolism, key genes involved in energy balance, lipid oxidation, and ketogenesis were examined. Compared to KD alone, PRE reduced the expression of *Ampk*, as well as *Cpt1a*, enzymes involved in lipid oxidation. In addition, PRE reduced the expression of *Hmgcs2*, the mitochondrial enzyme that catalyzes the first reaction of ketogenesis. Collectively, these changes suggest that PRE reduced the dependence of animals on ketones as a substrate. This may have been a result of elevated glucose levels and/or the increased production of microbial metabolites with PRE, including short-chain fatty acids as an alternative energy source. Unfortunately, short-chain fatty acids were not captured in our metabolomics screen.

Next, we examined the gut microbiota in reference, KDL, and KDL+PRE animals. While PRE is not digested by humans, it serves as a robust source of energy for the gut microbiota. Furthermore, there is common consensus that dietary intake of prebiotics in infant formula improves gut health by promoting beneficial microbes, specifically those within *Bifidobacterium* and *Lactobacillus* [58]. As anticipated, PRE inclusion resulted in increased microbial richness as calculated by the ACE index, as well as the relative abundances of *Bifidobacterium pesudolongum* and *Lactobacillus johnsonii*. Increases in richness have been previously observed with PRE consumption and have been linked to a more stable microbiota community [59]. However, a limitation of the study was 16S rRNA gene sequencing and the inability to infer microbial functionality from these data, as can be conducted with metagenomics.

To better understand the microbiota–gut–brain axis signals resulting from PRE supplementation in our study, we performed comprehensive metabolomics profiling on serum, liver, and the hippocampus. Multivariate PLS-DA demonstrated only modest separation between KDL and KDL+PRE treatments. Of note, PRE manipulation increased several metabolites in the hippocampus, including AMP, valine, glutathione, and CMP-*N*-acetyl-β-neuraminic acid, compounds that may be beneficial to brain function. Hippocampal glutathione and oxidative stress imbalances have been found to increase seizure severity in epileptic disorders [60]. An increase in glutathione in the hippocampus could confer a neuroprotective role by removing reactive oxygen species. As many of the implicated hippocampal metabolites were related to energy status and reactive oxygen species generation, we assessed mitochondrial respiration in the hippocampus. Compared to the normally developing reference group, we show the KD to increase CI+II activity, as well as maximal electron transport chain capacity. These results are in line with Ahn et al. [61], who demonstrated two weeks of the KD to improve both mitochondrial morphology and function in mouse neocortex. In addition, we show that basal and CI*_LEAK_* respiration were reduced with KD+PRE compared to KD alone, but no differences in any other respirometric measure. As previously mentioned, this may be due to the provision of alternative microbial fermentation substrates from PRE. Of note, certain fatty-acid-related species [62,63], as well as their metabolites, can cross the blood–brain barrier and are known to contribute to brain oxidative energy production [64].

## 5. Conclusions

The KD remains an efficacious and cost-effective option for drug-resistant epilepsy. In Canadian hospitals, initiation of the KD significantly decreased emergency department and inpatient visits, reducing direct inpatient costs by $1059 CDN per child per year when compared to age-matched children not on the diet [65]. In conclusion, results of the present study indicate that the inclusion of PRE did not impact KD-induced seizure protection. PRE had minor impacts on developmental milestones but enhanced the richness of the gut microbiota, including increases in *B. pseudolongum* and *L. johnsonii*. These microbial changes and their accompanying metabolites are likely responsible for the lower dependence of PRE animals on fatty acids and ketones as substrates. Overall, the results provide preclinical evidence showing PRE imparts microbial and metabolic benefits in the KD without impacting seizure mitigation in IS. This work is important and warrants future human clinical trials, as PRE inclusion may minimize the detrimental impacts of the KD on metabolism and the gut microbiota.

## Data Availability

The 16S rRNA gene sequence data supporting the conclusions of this article are deposited at the National Center for Biotechnology Information (BioProject PRJNA799481).

## Supplementary Materials

The following are available online at https://www.mdpi.com/article/10.3390/nu14112210/s1, Table S1: The formula ingredients, Table S2: Primers used in the present study, Table S3: The average relative abundances of operational taxonomic units detected in the present study.

## References

[1] Bergin A.M. (2017). Ketogenic Diet in Established Epilepsy Indications. Ketogenic Diet and Metabolic Therapies: Expanded Roles in Health and Disease.

[2] Kossoff E.H., Zupec-Kania B.A., Auvin S., Ballaban-Gil K.R., Christina Bergqvist A.G., Blackford R., Buchhalter J.R., Caraballo R.H., Cross J.H., Dahlin M.G. (2018). Optimal Clinical Management of Children Receiving Dietary Therapies for Epilepsy: Updated Recommendations of the International Ketogenic Diet Study Group. Epilepsia Open.

[3] Mu C., Choudhary A., Mayengbam S., Barrett K.T., Rho J.M., Shearer J., Scantlebury M.H. (2022). Seizure Modulation by the Gut Microbiota and Tryptophan-Kynurenine Metabolism in an Animal Model of Infantile Spasms. EBioMedicine.

[4] Mu C., Nikpoor N., Tompkins T.A., Choudhary A., Wang M., Marks W.N., Rho J.M., Scantlebury M.H., Shearer J. (2022). Targeted Gut Microbiota Manipulation Attenuates Seizures in a Model of Infantile Spasms Syndrome. JCI Insight.

[5] Paoli A., Mancin L., Bianco A., Thomas E., Mota J.F., Piccini F. (2019). Ketogenic Diet and Microbiota: Friends or Enemies?. Genes.

[6] Mu C., Shearer J., Scantlebury M.H., Marks W.N. (2022). The Ketogenic Diet and the Gut Microbiome. Ketogenic Diet and Metabolic Therapies: Expanded Roles in Health and Disease.

[7] Olson C.A., Vuong H.E., Yano J.M., Liang Q.X.Y., Nusbaum D.J., Hsiao E.Y. (2018). The Gut Microbiota Mediates the Anti-Seizure Effects of the Ketogenic Diet. Cell.

[8] Mu C., Nikpoor N., Tompkins T.A., Rho J.M., Scantlebury M.H., Shearer J. (2022). Probiotics Counteract Hepatic Steatosis Caused by Ketogenic Diet and Upregulate AMPK Signaling in a Model of Infantile Epilepsy. EBioMedicine.

[9] Mu C., Tompkins T.A., Rho J.M., Scantlebury M.H., Shearer J. (2022). Gut-Based Manipulations Spur Hippocampal Mitochondrial Bioenergetics in a Model of Pediatric Epilepsy. Biochim. et Biophys. Acta (BBA)—Mol. Basis Dis..

[10] Simm P.J., Bicknell-Royle J., Lawrie J., Nation J., Draffin K., Stewart K.G., Cameron F.J., Scheffer I.E., Mackay M.T. (2017). The Effect of the Ketogenic Diet on the Developing Skeleton. Epilepsy Res..

[11] Hong A.M., Turner Z., Hamdy R.F., Kossoff E.H. (2010). Infantile Spasms Treated with the Ketogenic Diet: Prospective Single-center Experience in 104 Consecutive Infants. Epilepsia.

[12] Arslan N., Guzel O., Kose E., Yılmaz U., Kuyum P., Aksoy B., Çalık T. (2016). Is Ketogenic Diet Treatment Hepatotoxic for Children with Intractable Epilepsy?. Seizure.

[13] Kang H.C., Lee Y.J., Lee J.S., Lee E.J., Eom S., You S.J., Kim H.D. (2011). Comparison of Short- versus Long-Term Ketogenic Diet for Intractable Infantile Spasms. Epilepsia.

[14] Dressler A., Benninger F., Trimmel-Schwahofer P., Groppel G., Porsche B., Abraham K., Muhlebner A., Samueli S., Male C., Feucht M. (2019). Efficacy and Tolerability of the Ketogenic Diet versus High-Dose Adrenocorticotropic Hormone for Infantile Spasms: A Single-Center Parallel-Cohort Randomized Controlled Trial. Epilepsia.

[15] Lyons L., Schoeler N.E., Langan D., Cross J.H. (2020). Use of Ketogenic Diet Therapy in Infants with Epilepsy: A Systematic Review and Meta-analysis. Epilepsia.

[16] Delzenne N.M., Neyrinck A.M., Cani P.D. (2013). Gut Microbiota and Metabolic Disorders: How Prebiotic Can Work?. Br. J. Nutr..

[17] Scantlebury M.H., Galanopoulou A.S., Chudomelova L., Raffo E., Betancourth D., Moshé S.L. (2010). A Model of Symptomatic Infantile Spasms Syndrome. Neurobiol. Dis..

[18] Panayiotopoulos C.P. (2010). Epileptic Encephalopathies in Infancy and Early Childhood. A Clinical Guide to Epileptic Syndromes and Their Treatment.

[19] Watanabe K. (1998). West Syndrome: Etiological and Prognostic Aspects. Brain Dev..

[20] Karvelas G., Lortie A., Scantlebury M.H., Duy P.T., Cossette P., Carmant L. (2009). A Retrospective Study on Aetiology Based Outcome of Infantile Spasms. Seizure.

[21] Wilmshurst J.M., Gaillard W.D., Vinayan K.P., Tsuchida T.N., Plouin P., van Bogaert P., Carrizosa J., Elia M., Craiu D., Jovic N.J. (2015). Summary of Recommendations for the Management of Infantile Seizures: Task Force Report for the ILAE Commission of Pediatrics. Epilepsia.

[22] Lux A.L., Edwards S.W., Hancock E., Johnson A.L., Kennedy C.R., Newton R.W., O’Callaghan F.J., Verity C.M., Osborne J.P., United Kingdom Infantile Spasms S. (2005). The United Kingdom Infantile Spasms Study (UKISS) Comparing Hormone Treatment with Vigabatrin on Developmental and Epilepsy Outcomes to Age 14 Months: A Multicentre Randomised Trial. Lancet Neurol..

[23] O’Callaghan F.J., Lux A.L., Darke K., Edwards S.W., Hancock E., Johnson A.L., Kennedy C.R., Newton R.W., Verity C.M., Osborne J.P. (2011). The Effect of Lead Time to Treatment and of Age of Onset on Developmental Outcome at 4 Years in Infantile Spasms: Evidence from the United Kingdom Infantile Spasms Study. Epilepsia.

[24] Closa-Monasterolo R., Gispert-Llaurado M., Luque V., Ferre N., Rubio-Torrents C., Zaragoza-Jordana M., Escribano J. (2013). Safety and Efficacy of Inulin and Oligofructose Supplementation in Infant Formula: Results from a Randomized Clinical Trial. Clin. Nutr..

[25] Vandenplas Y., de Greef E., Veereman G. (2014). Prebiotics in Infant Formula. Gut Microbes..

[26] Choudhary A., Mu C., Barrett K., Williams-Dyjur C., Marks W.N., Shearer J., Rho J.M., Scantlebury M.H. (2021). The Link between Brain Acidosis, Breathing, and Seizures: A Novel Mechanism of Action for the Ketogenic Diet in a Model of Infantile Spasms. Brain Commun..

[27] Ruhela R.K., Soni S., Sarma P., Prakash A., Medhi B. (2019). Negative Geotaxis: An Early Age Behavioral Hallmark to VPA Rat Model of Autism. Ann. Neurosci..

[28] Shearer J., Fueger P.T., Vorndick B., Bracy D.P., Rottman J.N., Clanton J.A., Wasserman D.H. (2004). AMP Kinase-Induced Skeletal Muscle Glucose but Not Long-Chain Fatty Acid Uptake Is Dependent on Nitric Oxide. Diabetes.

[29] Kozich J.J., Westcott S.L., Baxter N.T., Highlander S.K., Schloss P.D. (2013). Development of a Dual-Index Sequencing Strategy and Curation Pipeline for Analyzing Amplicon Sequence Data on the MiSeq Illumina Sequencing Platform. Appl. Environ. Microbiol..

[30] Hughey C.C., Ma L., James F.D., Bracy D.P., Wang Z., Wasserman D.H., Rottman J.N., Hittel D.S., Shearer J. (2013). Mesenchymal Stem Cell Transplantation for the Infarcted Heart: Therapeutic Potential for Insulin Resistance beyond the Heart. Cardiovasc. Diabetol..

[31] Newell C., Sabouny R., Hittel D.S., Shutt T.E., Khan A., Klein M.S., Shearer J. (2018). Mesenchymal Stem Cells Shift Mitochondrial Dynamics and Enhance Oxidative Phosphorylation in Recipient Cells. Front. Physiol..

[32] Herbst E.A., Holloway G.P. (2015). Permeabilization of Brain Tissue in Situ Enables Multiregion Analysis of Mitochondrial Function in a Single Mouse Brain. J. Physiol..

[33] Southam A.D., Weber R.J., Engel J., Jones M.R., Viant M.R. (2016). A Complete Workflow for High-Resolution Spectral-Stitching Nanoelectrospray Direct-Infusion Mass-Spectrometry-Based Metabolomics and Lipidomics. Nat. Protoc..

[34] Lai Y.-S., Chen W.-C., Kuo T.-C., Ho C.-T., Kuo C.-H., Tseng Y.J., Lu K.-H., Lin S.-H., Panyod S., Sheen L.-Y. (2015). Mass-Spectrometry-Based Serum Metabolomics of a C57BL/6J Mouse Model of High-Fat-Diet-Induced Non-Alcoholic Fatty Liver Disease Development. J. Agric. Food Chem..

[35] Mayengbam S., Chleilat F., Reimer R.A. (2020). Dietary Vitamin B6 Deficiency Impairs Gut Microbiota and Host and Microbial Metabolites in Rats. Biomedicines.

[36] Tautenhahn R., Patti G.J., Rinehart D., Siuzdak G. (2012). XCMS Online: A Web-Based Platform to Process Untargeted Metabolomic Data. Anal. Chem..

[37] Smith C.A., O’Maille G., Want E.J., Qin C., Trauger S.A., Brandon T.R., Custodio D.E., Abagyan R., Siuzdak G. (2005). METLIN: A Metabolite Mass Spectral Database. Ther. Drug Monit..

[38] Wishart D.S., Jewison T., Guo A.C., Wilson M., Knox C., Liu Y., Djoumbou Y., Mandal R., Aziat F., Dong E. (2013). HMDB 3.0—The Human Metabolome Database in 2013. Nucleic Acids Res..

[39] Pang Z., Chong J., Zhou G., de Lima Morais D.A., Chang L., Barrette M., Gauthier C., Jacques P.-É., Li S., Xia J. (2021). MetaboAnalyst 5.0: Narrowing the Gap between Raw Spectra and Functional Insights. Nucleic Acids Res..

[40] Kim D.Y., Rho J.M. (2008). The Ketogenic Diet and Epilepsy. Curr. Opin. Clin. Nutr. Metab. Care.

[41] Peng M., Tabashsum Z., Patel P., Bernhardt C., Biswas D. (2018). Linoleic Acids Overproducing Lactobacillus Casei Limits Growth, Survival, and Virulence of Salmonella Typhimurium and Enterohaemorrhagic Escherichia Coli. Front. Microbiol..

[42] Wright A.T. (2019). Gut Commensals Make Choline Too. Nat. Microbiol..

[43] Simeone T.A., Simeone K.A., Stafstrom C.E., Rho J.M. (2018). Do Ketone Bodies Mediate the Anti-Seizure Effects of the Ketogenic Diet?. Neuropharmacology.

[44] Styr B., Gonen N., Zarhin D., Ruggiero A., Atsmon R., Gazit N., Braun G., Frere S., Vertkin I., Shapira I. (2019). Mitochondrial Regulation of the Hippocampal Firing Rate Set Point and Seizure Susceptibility. Neuron.

[45] Gibson G.R., Hutkins R., Sanders M.E., Prescott S.L., Reimer R.A., Salminen S.J., Scott K., Stanton C., Swanson K.S., Cani P.D. (2017). Expert Consensus Document: The International Scientific Association for Probiotics and Prebiotics (ISAPP) Consensus Statement on the Definition and Scope of Prebiotics. Nat. Rev. Gastroenterol. Hepatol..

[46] Zupec-Kania B.A., Aldaz V., Montgomery M.E., Kostas K.C. (2011). Enteral and Parenteral Applications of Ketogenic Diet Therapy: Experiences from Four Centers. ICAN Infant Child Adolesc. Nutr..

[47] Janicot R., Shao L., Stafstrom C.E. (2021). 2-deoxyglucose and Β-hydroxybutyrate Fail to Attenuate Seizures in the Betamethasone-NMDA Model of Infantile Spasms. Epilepsia Open.

[48] Yum M.-S., Lee M., Woo D.-C., Kim D.W., Ko T.-S., Velíšek L. (2015). β-Hydroxybutyrate Attenuates NMDA-Induced Spasms in Rats with Evidence of Neuronal Stabilization on MR Spectroscopy. Epilepsy Res..

[49] Amadieu C., Coste V., Neyrinck A.M., Thijssen V., Leyrolle Q., Bindels L.B., Piessevaux H., Starkel P., de Timary P., Delzenne N.M. (2022). Restoring an Adequate Dietary Fiber Intake by Inulin Supplementation: A Pilot Study Showing an Impact on Gut Microbiota and Sociability in Alcohol Use Disorder Patients. Gut Microbes..

[50] Nettleton J.E., Klancic T., Schick A., Choo A.C., Cheng N., Shearer J., Borgland S.L., Rho J.M., Reimer R.A. (2021). Prebiotic, Probiotic, and Synbiotic Consumption Alter Behavioral Variables and Intestinal Permeability and Microbiota in BTBR Mice. Microorganisms.

[51] Coffey K.R., Marx R.G., Neumaier J.F. (2019). DeepSqueak: A Deep Learning-Based System for Detection and Analysis of Ultrasonic Vocalizations. Neuropsychopharmacology.

[52] Paul H.A., Collins K.H., Nicolucci A.C., Urbanski S.J., Hart D.A., Vogel H.J., Reimer R.A. (2019). Maternal Prebiotic Supplementation Reduces Fatty Liver Development in Offspring through Altered Microbial and Metabolomic Profiles in Rats. FASEB J..

[53] Reid D.T., Eller L.K., Nettleton J.E., Reimer R.A. (2016). Postnatal Prebiotic Fibre Intake Mitigates Some Detrimental Metabolic Outcomes of Early Overnutrition in Rats. Eur. J. Nutr..

[54] Loman B.R., Hernandez-Saavedra D., An R., Rector R.S. (2018). Prebiotic and Probiotic Treatment of Nonalcoholic Fatty Liver Disease: A Systematic Review and Meta-Analysis. Nutr. Rev..

[55] Parnell J.A., Raman M., Rioux K.P., Reimer R.A. (2012). The Potential Role of Prebiotic Fibre for Treatment and Management of Non-Alcoholic Fatty Liver Disease and Associated Obesity and Insulin Resistance. Liver Int..

[56] Beisner J., Rosa L.F., Kaden-Volynets V., Stolzer I., Gunther C., Bischoff S.C. (2021). Prebiotic Inulin and Sodium Butyrate Attenuate Obesity-Induced Intestinal Barrier Dysfunction by Induction of Antimicrobial Peptides. Front. Immunol..

[57] Bomhof M.R., Paul H.A., Geuking M.B., Eller L.K., Reimer R.A. (2016). Improvement in Adiposity with Oligofructose Is Modified by Antibiotics in Obese Rats. FASEB J..

[58] Mayengbam S., Lambert J.E., Parnell J.A., Tunnicliffe J.M., Nicolucci A.C., Han J., Sturzenegger T., Shearer J., Mickiewicz B., Vogel H.J. (2019). Impact of Dietary Fiber Supplementation on Modulating Microbiota-Host-Metabolic Axes in Obesity. J. Nutr. Biochem..

[59] Tap J., Furet J.P., Bensaada M., Philippe C., Roth H., Rabot S., Lakhdari O., Lombard V., Henrissat B., Corthier G. (2015). Gut Microbiota Richness Promotes Its Stability upon Increased Dietary Fibre Intake in Healthy Adults. Environ. Microbiol..

[60] Cardenas-Rodriguez N., Coballase-Urrutia E., Perez-Cruz C., Montesinos-Correa H., Rivera-Espinosa L., Sampieri A., Carmona-Aparicio L. (2014). Relevance of the Glutathione System in Temporal Lobe Epilepsy: Evidence in Human and Experimental Models. Oxid. Med. Cell. Longev..

[61] Ahn Y., Sabouny R., Villa B.R., Yee N.C., Mychasiuk R., Uddin G.M., Rho J.M., Shutt T.E. (2020). Aberrant Mitochondrial Morphology and Function in the BTBR Mouse Model of Autism Is Improved by Two Weeks of Ketogenic Diet. Int. J. Mol. Sci..

[62] Andersen J.V., Westi E.W., Jakobsen E., Urruticoechea N., Borges K., Aldana B.I. (2021). Astrocyte Metabolism of the Medium-Chain Fatty Acids Octanoic Acid and Decanoic Acid Promotes GABA Synthesis in Neurons via Elevated Glutamine Supply. Mol. Brain.

[63] Spector R. (1988). Fatty-Acid Transport through the Blood-Brain Barrier. J. Neurochem..

[64] Ebert D., Haller R.G., Walton M.E. (2003). Energy Contribution of Octanoate to Intact Rat Brain Metabolism Measured by 13C Nuclear Magnetic Resonance Spectroscopy. J. Neurosci..

[65] Whiting S., Donner E., RamachandranNair R., Grabowski J., Jette N., Duque D.R. (2017). Decreased Health Care Utilization and Health Care Costs in the Inpatient and Emergency Department Setting Following Initiation of Ketogenic Diet in Pediatric Patients: The Experience in Ontario, Canada. Epilepsy Res..

