# Supplementary material for: Addition of Prebiotics to the Ketogenic Diet Improves Metabolic Profile but Does Not Affect Seizures in a Rodent Model of Infantile Spasms Syndrome
Source: Nutrients. 2022 May 26;14(11):2210. doi: 10.3390/nu14112210 (PMC9182787; doi:10.3390/nu14112210)
Supplement: Supplementary file 1 [file nutrients-14-02210-s001.zip › nutrients-1702438-supplementary.pdf]

Supplemental Materials

Addition of Prebiotics to the Ketogenic Diet Improves Metabolic Profile but Does Not Affect Seizures in a Rodent Model of Infantile Spasms Syndrome

Chunlong Mu [1,*], Angela Pochakom [1], Raylene A. Reimer [1,2,3], Anamika Choudhary [3,4], Melinda Wang [3], Jong M. Rho [5], Morris H. Scantlebury [3,4,6] and Jane Shearer [1,2,3]

[1] Department of Biochemistry and Molecular Biology, Cumming School of Medicine, University of Calgary, 3330 Hospital Drive NW, Calgary, AB T2N 4N1, Canada; angela.pochakom@ucalgary.ca (A.P.); reimer@ucalgary.ca (R.A.R.); jshearer@ucalgary.ca (J.S.)

[2] Faculty of Kinesiology, University of Calgary, 2500 University Drive NW, Calgary, AB T2N 1N4, Canada

[3] Alberta Children's Hospital Research Institute, Cumming School of Medicine, University of Calgary, 3330 Hospital Drive NW, Calgary, AB T2N 4N1, Canada; melinda.wang@ucalgary.ca

[4] Department of Pediatrics, Cumming School of Medicine, University of Calgary, 3330 Hospital Drive NW, Calgary, AB T2N 4N1, Canada; anamika.choudhary@ucalgary.ca (A.C.); mor-ris.scantlebury@albertahealthservices.ca (M.H.S.)

[5] Departments of Neurosciences, Pediatrics and Pharmacology, University of California San Diego , 9500 Gil-man Drive, La Jolla, CA 92093, USA; jrho@health.ucsd.edu

[6] Department of Clinical Neurosciences, Hotchkiss Brain Institute, Cumming School of Medicine,

University of Calgary, 3330 Hospital Drive NW, Calgary, AB T2N 4N1, Canada

*Correspondence: chunlong.mu1@ucalgary.ca

Table S1. Detailed composition of the ketogenic diet. Diets are based on Nutricia KetoCal Formulation (see: https://shop.myketocal.com/product/ketocal-41-powder) for detailed information.

**Ingredients**	**Ketogenic Rat Formula Amount (g/Kg)**	**Ingredients**	**Ketogenic Rat Formula Amount (g/Kg)**
**Calories Total**	2657 kcal	**Vitamin and Mineral Composition**	
Fat Total	264.75	Sodium	0.39
Saturated	31.23	Calcium	43.15
+Trans	0	Iron	0.003
Polyunsaturated	129.84	Linoleic acid	2.85
Omega-6	168.79	Aspartame	0.015
Omega-3	2.60	Thiamine	0.00019
Monounsaturated	64.92	Riboflavin	0.00020
Cholesterol	0.11	Niacin	0.0023
Carbohydrate	20.41	Folic Acid	5.88E-05
Fibre	0	Pantothenic Acid	0.00080
Sugars	5.79	Biotin	3.87E-06
Protein	55.83	Choline	0.15
Alanine	6.91	Inositol	0.042
Arginine	4.54	Phosphorus	0.20
Aspartic Acid	16.58	Magnesium	0.034
Cysteine	3.36	Zinc	0.0018
Glutamic Acid	26.25	Iodine	2.54E-05
Glycine	2.76	Manganese	0.00050
Histidine	3.16	Copper	0.00025
Isoleucine BCAA	9.08	Molybdenum	8.05E-06
Leucine BCAA	17.37	Chromium	5.88E-06
Lysine EAA	3.16	Selenium	1.05E-05
Phenylalanine EAA	5.13	Potassium	0.33
Proline	13.03	Chloride	0.15
Serine	9.08	Vitamin A	464.32 IU
Threonine EAA	13.03	Vitamin C	23.16 %DV + 0.018g
Tryptophan EAA	3.75	Vitamin B6	0.00020
Tyrosine	5.33	Vitamin B12	4.024E-07
Valine EAA BCAA	10.26	Vitamin D	34.74 %DV
		Vitamin D3	64.39 IU
		Vitamin E	259.67 %DV + 3.40 IU
		Vitamin K	1.24E-05

Table S2 Primers used in the present study.

Target	Forward	Reverse
Fas	GACCCTGACTCCAAGTTATTCGA	CGTCAAGCGGGAGACAGACT
Cpt1a	GCTTCCCCTTACTGGTTCC	AACTGGCAGGCAATGAGACT
Ampk	AGATATCAGGGAACATGAATGG	ATGGTTGAACTATAAGACGGG
Acat1	ATCTCAGTGAAAGGTAAACCAG	CTTTGGCACTTTACTGAAGTC
Hmgcs2	CAGCTTACCGCAGGAAAATCC	CAAAAGGGTGTGTGGAAGATCA
Ywhaz	TTGAGCAGAAGACGGAAGGT	GAAGCATTGGGGATCAAGAA
CycA	AGCACTGGGGAGAAAGGATT	AGCCACTCAGTCTTGGCAGT

Table S3 The average relative abundances of operational taxonomic units (OTUs) detected in the present study. R - normally developing reference group; KDL - ketogenic diet formula; KDL+PRE - ketogenic diet formula plus prebiotic fiber

OTU ID	Closest species	R	KDL	KDL+PRE	P value
Otu001	*Ligilactobacillus animalis*	68.866a	20.641b	3.675c	<0.001
Otu002	*Enterococcus* unclassified	0.319a	22.143b	29.848b	<0.001
Otu003	*Aggregatibacter pneumotropica*	6.490a	17.468ab	23.839b	0.014
Otu004	*Escherichia coli*	7.757	12.757	17.397	0.496
Otu005	*Streptococcus acidominimus*	3.374a	11.599b	8.660ab	0.004
Otu006	*Lactobacillus johnsonii*	9.927a	1.982b	7.237a	0.001
Otu007	*Lactobacillus reuteri*	1.593	2.413	4.556	0.091
Otu008	*Staphylococcus* unclassified	0.315a	2.389b	3.630b	0.001
Otu009	*Planococcaceae* unclassified	0.000a	6.874b	0.000a	0.002
Otu010	*Acinetobacter guillouiae*	0.000a	0.873b	0.565b	0.001
Otu011	*Romboutsia timonensis*	0.812a	0.000b	0.000b	<0.001
Otu012	Bacillales unclassified	0.000a	0.100b	0.097b	0.003
Otu013	*Lactococcus lactis*	0.000a	0.130a	0.034b	0.001
Otu015	*Rothia nasimurium*	0.004	0.118	0.007	0.414
Otu017	*Lactobacillales* unclassified	0.028	0.014	0.046	0.258
Otu018	*Streptococcus* danieliae	0.085a	0.000b	0.016ab	0.003
Otu019	*Flavobacteriaceae* unclassified	0.000a	0.053b	0.007a	0.001
Otu020	*Allobacillus halotolerans*	0.000a	0.053b	0.005a	0.001
Otu021	SUP05 unclassified	0.041	0.006	0.014	0.114
Otu023	*Staphylococcuss sciuri*	0.000	0.012	0.034	0.124
Otu024	*Ligilactobacillus pobuzihii*	0.000a	0.028b	0.021ab	0.014
Otu025	*Ligilactobacillus faecis*	0.004ab	0.035a	0.000b	0.006
Otu026	*Lactobacillus* unclassified	0.039a	0.010b	0.002b	0.001
Otu027	*Streptococcus* unclassified	0.000	0.004	0.011	0.104
Otu028	*Lactobacillales* unclassified	0.000a	0.000a	0.020b	0.006
Otu029	*Streptococcus luteciae*	0.000a	0.012ab	0.014b	0.017
Otu030	*Lactobacillus* unclassified	0.000a	0.000a	0.016b	0.018
Otu031	*Microbacterium lacticum*	0.000	0.004	0.020	0.223
Otu032	*Bacteroides vulgatus*	0.010	0.006	0.004	0.466
Otu033	*Lactobacillales* unclassified	0.000	0.016	0.016	0.056
Otu035	*Lactobacillus* unclassified	0.006	0.000	0.004	0.195
Otu036	*Bacteroidess acidifaciens*	0.002	0.002	0.007	0.434
Otu038	*Bacteroidales* S24-7 unclassified	0.004	0.004	0.005	0.901
Otu039	*Lactobacillales* unclassified	0.000a	0.000a	0.011b	0.018
Otu041	*Enterobacteriaceae* unclassified	0.000	0.008	0.005	0.241
Otu042	*Akkermansia muciniphila*	0.006	0.000	0.005	0.178
Otu043	*Enterococcus saccharolyticus*	0.000	0.004	0.007	0.196
Otu044	*Lachnospiraceae* unclassified	0.006	0.008	0.002	0.275
Otu045	*Lactobacillus* unclassified	0.004	0.008	0.000	0.164
Otu046	*Bifidobacterium pseudolongum*	0.000a	0.000a	0.013b	0.005
Otu047	*Lactobacillus* unclassified	0.000a	0.000a	0.014b	0.018
Otu049	*Aggregatibacter pneumotropica*	0.000	0.004	0.004	0.345
Otu050	*Blautia wexlerae*	0.004	0.002	0.005	0.615
Otu056	*Faecalibacterium rausnitzii*	0.006	0.002	0.004	0.417

Note: Different letters within a row indicated a significance (p<0.05) between groups.
